# Purpura as the initial manifestation of IgG4-related disease with concomitant systemic lupus erythematosus: a case report

**DOI:** 10.3389/fmed.2026.1872392

**Published:** 2026-06-22

**Authors:** Yi Wei, Fuhua Chen, Ruomeng Li, Zhen Zhao, Yajuan Huang

**Affiliations:** 1 Department of Nephrology, The Sixth Affiliated Hospital, Sun Yat-sen University, Guangzhou, Guangdong, China; 2 Biomedical Innovation Center, The Sixth Affiliated Hospital, Sun Yat-sen University, Guangzhou, Guangdong, China

**Keywords:** purpura, IgG4-related disease, systemic lupus erythematosus, IgG4-related kidney disease, lupus nephritis

## Abstract

Purpura is a common clinical manifestation of a wide variety of diseases, including extremely uncommon conditions. In this study, we report the case of a 61-year-old man who initially presented with purpura and was ultimately diagnosed with coexisting immunoglobulin G4 (IgG4)-related tubulointerstitial nephritis (TIN) and class II lupus nephritis. The patient was treated with a combination of prednisone (Pred), hydroxychloroquine (HCQ), and mycophenolate mofetil (MMF). Within 1 month of treatment, the purpura resolved, and qualitative urine protein testing turned negative. During an 8-month follow-up period, sustained clinical improvement was observed. For the first time, this study highlights purpura as the initial presentation of concurrent IgG4-related disease and systemic lupus erythematosus (SLE) and describes a novel renal pathological overlap characterized by IgG4-related tubulointerstitial injury alongside lupus-induced mesangioproliferative glomerulonephritis (MsPGN). These findings provide new insights into the diagnosis, management, and prognosis of this rare condition.

## Introduction

Purpura is a common manifestation of cutaneous hemorrhage, characterized by non-blanching red or purple spots measuring 3–10 mm, with a predilection for the lower extremities. Its pathogenesis includes platelet deficiency or dysfunction, vascular disorders, and coagulation dysfunction. As a non-specific clinical presentation, purpura is associated with a wide variety of diseases, including immune-related disorders (1, 2). Consequently, elucidating the complex etiology of this manifestation can be challenging, particularly when purpura is the initial presentation of the disease.

Immunoglobulin G4-related disease (IgG4-RD) is a systemic, immune-mediated fibro-inflammatory disorder characterized by elevated serum IgG4 levels and organ enlargement (3). Histopathological features of IgG4-RD include the infiltration of IgG4^+^ plasma cells, storiform fibrosis, and obliterative phlebitis. Glucocorticoids rapidly improve clinical symptoms and remain the first-line therapy, although regular follow-up is required during long-term management to monitor drug-related adverse effects.

Systemic lupus erythematosus (SLE) is a systemic autoimmune disease characterized by the presence of specific autoimmune antibodies. The kidneys are among the most commonly involved organs in SLE; therefore, renal biopsy plays a vital role in the diagnosis of the disease. Traditional therapeutic strategies include glucocorticoids, immunosuppressants, and biologics. More recently, emerging therapeutic methods, such as mesenchymal stem cell therapy (4) and chimeric antigen receptor T (CAR-T) cell therapy (5), have provided new insights into the pathogenesis and management of SLE.

In this study, we report a rare comorbidity in which purpura served as the initial manifestation of concurrent IgG4-RD and SLE, highlighting the clinical and pathological features of this rare overlap syndrome. We further review the previous studies and explore potential mechanistic associations between these two immune-mediated diseases.

## Case report

### Patient concerns

We report the case of a 61-year-old man who presented to the dermatology outpatient clinic with a 6-month history of symmetric purpura involving both lower extremities. He denied accompanying symptoms, including fever, arthralgia, Raynaud’s phenomenon, and weight loss. He had been diagnosed with immune thrombocytopenia in 2023 based on bone marrow morphology examination, and his platelet count returned to normal levels after treatment with platelet transfusion and thrombopoietin. He also had a 10-year history of hypertension and had been taking amlodipine besylate and metoprolol irregularly. Family history was non-contributory.

Laboratory examinations revealed normal platelet count and normal coagulation function, but persistent hematuria and proteinuria. Therefore, the patient was referred to the nephrology outpatient clinic. Further investigation revealed that the urine protein-to-creatinine ratio (PCR) was 1,269.75 mg/gCr, and the urine albumin-to-creatinine ratio (ACR) was 113.43 mg/gCr. He was suspected of having IgA vasculitis and was treated with valsartan, an angiotensin receptor blocker (ARB). However, no significant reduction was observed in proteinuria after 1 month of treatment. He subsequently consented to a renal biopsy and was admitted to the Department of Nephrology for further evaluation and management.

### Clinical investigation and diagnosis

**Figure 1:**
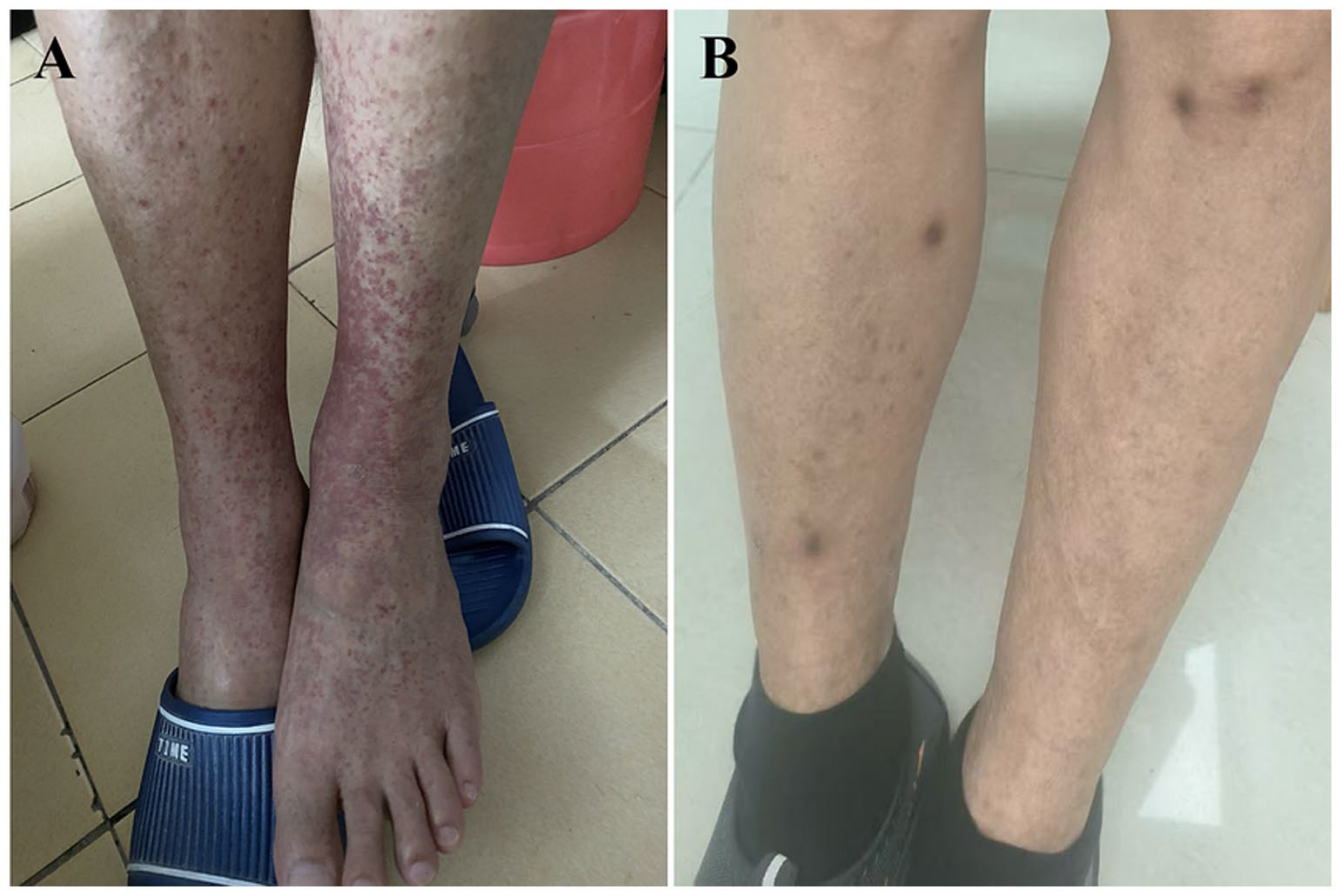
Purpura was the initial manifestation in this case of IgG4-RD overlapping with SLE. **(A)** Purpura was observed at the patient’s first visit to the nephrology outpatient clinic. **(B)** The purpura resolved after 1 month of treatment with prednisone, MMF, and HCQ.

On admission, his body mass index was 21.23 kg/m^2^, and his blood pressure was 159/84 mmHg. Physical examination revealed symmetrical, non-blanching purpura on both lower extremities, some of which were partially confluent (Figure 1A). Laboratory examination revealed mild anemia, urinary abnormalities, and impaired liver function, along with increased serum *κ* and *λ* light chain levels (Supplementary Table S1). No evidence of current hepatitis B, hepatitis C, or tuberculosis infection was found.

Further evaluation revealed hypocomplementemia and positivity for anti-nuclear antibody (ANA), anti-nucleosome antibody (ANuA), and the direct antiglobulin test (DAT), whereas anti-double-stranded DNA (dsDNA) antibody and anti-Smith (Sm) antibody were negative. Notably, he also exhibited hypergammaglobulinemia with a significantly elevated serum IgG4 level (Table 1). Computed tomography (CT) of the head, chest, and abdomen demonstrated diffuse renal enlargement (Supplementary Figure S1).

**Table 1:** Immunity parameters.

Parameters	Value	Reference range
IgG (g/L)	98	7–15
IgA (g/L)	1.19	0.7–4
IgM (g/L)	0.41	0.4–2.6
C3 (g/L)	0.47	0.79–1.52
C4 (g/L)	0.03	0.2–0.4
IgG4 (g/L)	60.24	Not more than 2
RF (IU/mL)	2.76	0–14
Anti-CCP (U/mL)	3.8	0–5
ANA	1:1000	Negative
Anti-Sm	Negative	Negative
Anti-Sm/RNP	Negative	Negative
Anti-SSA	Negative	Negative
Anti-SSB	Negative	Negative
ANuA	Positive	Negative
Anti-dsDNA (IU/mL)	3.26	Less than 20
DAT	Positive	Negative
LA	0.85	Less than 1.2
ACA (U/mL)	11.74	Less than 20
β2-GP1-Ab (U/mL)	18.62	Less than 20
MPO-Ab	Negative	Negative
PR3-Ab	Negative	Negative
pANCA	Negative	Negative
cANCA	Negative	Negative
Anti-PLA2R Ab (RU/mL)	1.88	Less than 22

**Figure 2:**
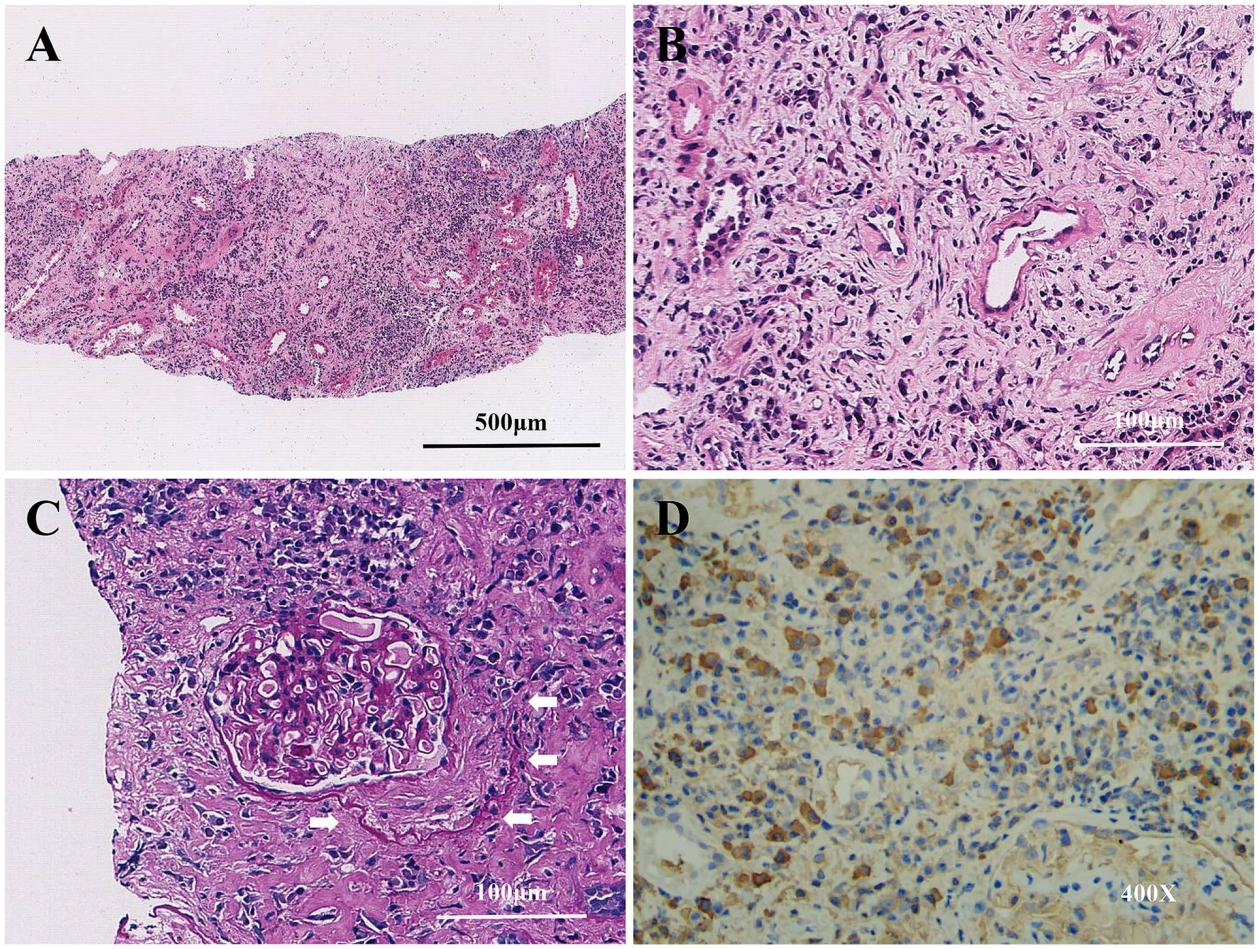
Renal biopsy demonstrates coexisting IgG4-related TIN with class II lupus nephritis. **(A)** Hematoxylin and eosin (H&E) staining shows abundant inflammatory cell infiltration in the renal interstitium, predominantly composed of plasma cells, lymphocytes, and monocytes. **(B)** H&E staining demonstrates focal storiform fibrosis. **(C)** Periodic acid–Schiff staining reveals pseudocrescent (white arrow) and mild-to-moderate mesangial cell proliferation with the expansion of mesangial matrix. **(D)** Immunohistochemical staining reveals more than 30 interstitial IgG4^+^ cells per HPF.

Thus, a renal biopsy was performed. Light microscopy revealed severe acute and chronic interstitial lesions with abundant infiltration of plasma cells, lymphocytes, and monocytes (Figure 2A). Focal storiform fibrosis (Figure 2B) and pseudocrescents (Figure 2C, white arrow) were also identified. In addition, global glomerulosclerosis was detected in 5 of 15 examined glomeruli, with mild-to-moderate proliferation of mesangial cells and expansion of mesangial matrix in the remaining glomeruli. No definite arteritis or phlebitis was observed. Arterial wall thickening, intimal fibrosis, and luminal narrowing were noted. Immunofluorescence microscopy revealed mesangial deposition of immune complexes, including IgG1 (3+), IgG2 (1+), IgG3 (1+), IgG4 (1+), IgA (1+), IgM (1+), complement component 3 (C3, 3+), C1q (±), *κ* (2+), and *λ* (3+). However, fibrinogen, albumin, phospholipase A2 receptor (PLA2R), thrombospondin type-1 domain-containing 7A (THSD7A), and hepatitis C virus (HCV) antigens were not detected. Electron microscopy showed electron-dense deposits within the mesangium but not along the tubular basement membrane, accompanied by podocyte foot process effacement (51–75%). Immunohistochemical staining (Figure 2D) showed more than 30 interstitial IgG4^+^ cells per high-power field (HPF), with an IgG4^+^/IgG^+^ cell ratio exceeding 40%. Congo red staining was negative.

Based on the clinical manifestations, laboratory data, and histopathological findings, a diagnosis of IgG4-RD overlapping with SLE was established according to the 2019 european league against rheumatism/American college of rheumatology (EULAR/ACR) classification criteria for IgG4-RD (6) and the 2019 EULAR/ACR classification criteria for SLE (7) (Supplementary Table S2). Meanwhile, a diagnosis of IgG4-related kidney disease (IgG4-RKD) was established according to the 2020 diagnostic criteria of IgG4-RKD (8), and a diagnosis of mesangioproliferative glomerulonephritis (MsPGN)/class II lupus nephritis was established according to the 2018 international society of nephrology/renal pathology society (ISN/RPS) classification for lupus nephritis (9).

### Treatment and follow-up

**Figure 3:**
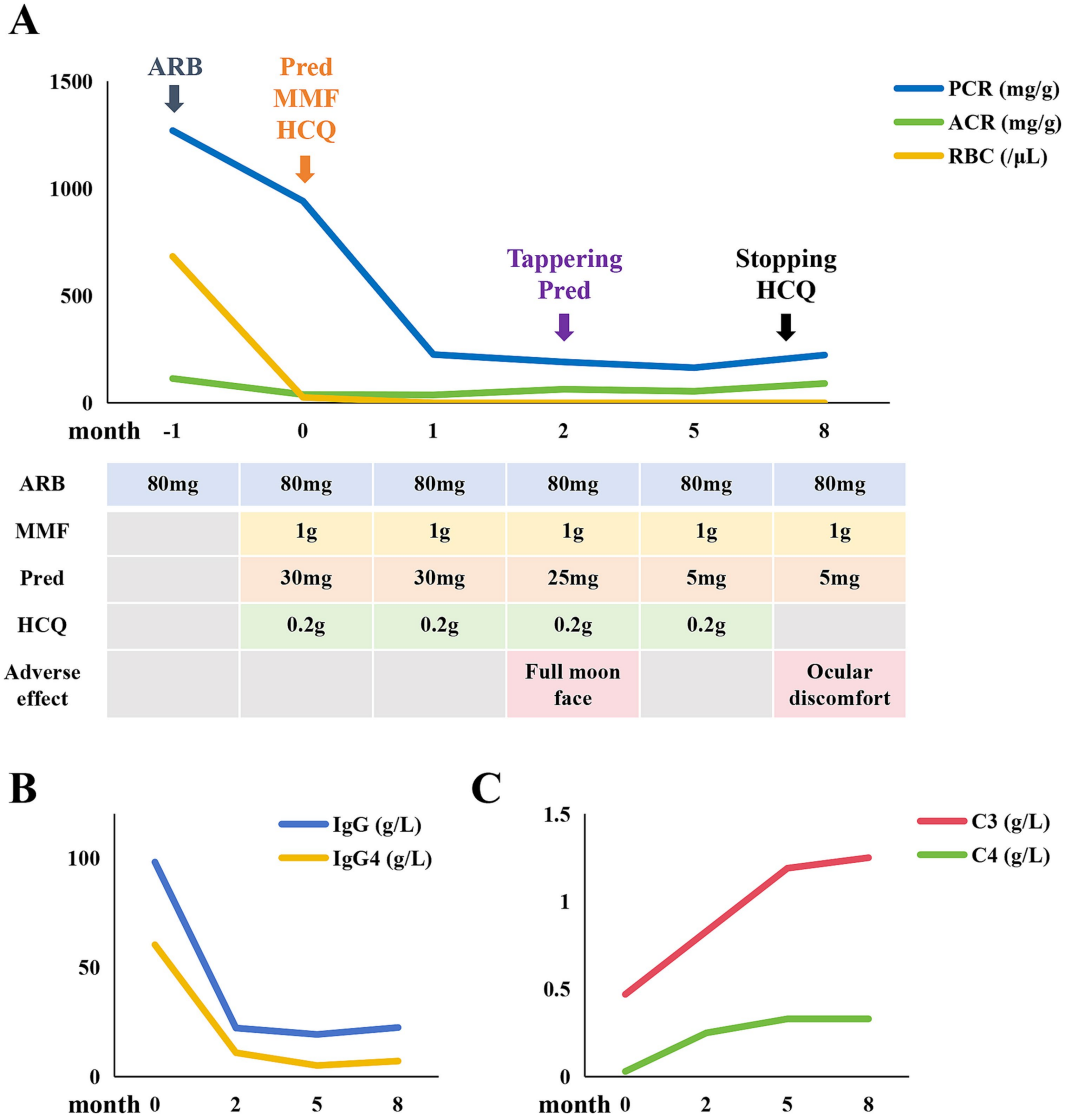
Treatment with prednisone, HCQ, and MMF achieved sustained clinical improvement over an 8-month follow-up period. **(A)** Proteinuria and hematuria improved during the 8-month treatment. Medication dosages were adjusted due to the drug-related adverse effects. **(B)** Serum IgG and IgG4 levels decreased during follow-up. **(C)** Serum C3 and C4 levels increased during follow-up.

The patient was started on prednisone (Pred, 0.5 mg/kg/day), hydroxychloroquine (HCQ, 200 mg/day), and mycophenolate mofetil (MMF, 1 g/day). After 1 month of treatment, the purpura resolved (Figure 1B), and no hematuria or proteinuria was detected by the urinalysis (Figure 3A). At 2 months, his liver function improved, and no deterioration in renal function was observed (Supplementary Figure S2). In addition, serum IgG levels decreased from 98.00 g/L to 22.21 g/L, and IgG4 levels decreased from 60.24 g/L to 10.93 g/L (Figure 3B), whereas C3 levels increased from 0.47 g/L to 0.83 g/L, and C4 levels increased from 0.03 g/L to 0.25 g/L (Figure 3C). However, the patient developed full moon face, suggesting glucocorticoid-related adverse effects. Prednisone was therefore tapered by 5 mg per week to 15 mg/day, followed by a gradual reduction of 5 mg per month.

After prednisone tapering, disease activity and medication-related adverse events were continuously monitored. At 5 months, sustained resolution of proteinuria and hematuria was observed, accompanied by continued improvement in IgG, IgG4, C3, and C4 levels. At 7 months, hydroxychloroquine was discontinued due to ocular discomfort. During the 8-month follow-up period, the patient showed sustained clinical improvement, with a PCR of less than 300 mg/gCr, resolved hematuria, decreased IgG levels (22.45 g/L), decreased IgG4 levels (7.13 g/L), increased C3 levels (1.25 g/L), and increased C4 levels (0.33 g/L). He remains under close follow-up to monitor disease activity and potential treatment-related adverse effects.

## Discussion

To the best of our knowledge, this is the first reported case in which purpura served as the initial manifestation of concomitant IgG4-RD and SLE. A common clinical presentation may conceal uncommon conditions, highlighting the importance of a thorough differential diagnosis.

Cutaneous involvement in IgG4-RD is rare, with a prevalence of 1.6–4.2% (10). Approximately 70% of patients with skin involvement exhibit additional organ involvement. However, both purpura and renal involvement are particularly rare in IgG4-RD with cutaneous involvement. Tokura et al. (11) have classified IgG4-RD-associated skin manifestations into seven subtypes, among which purpura is considered attributable to hypergammaglobulinemia with leukocytoclastic vasculitis as an indirect presentation rather than as a direct consequence of IgG4^+^ plasma cell infiltration.

In contrast, cutaneous manifestations are common in SLE. Purpura is relatively common in SLE and is attributed to thrombocytopenia, vasculitis, and other mechanisms. Cutaneous vasculitis is frequent in SLE, with an incidence of 10–20%, and leukocytoclastic vasculitis represents the most common (64%) histopathological pattern (12, 13). Among SLE patients with cutaneous vasculitis, 25% present with palpable purpura and 3% develop renal involvement.

We speculated that purpura in this case might be caused by leukocytoclastic vasculitis; however, the patient’s unwillingness to undergo skin biopsy precluded confirmation of the underlying pathogenesis. In this case, purpura improved after 1 month of treatment with no recurrence, in parallel with systemic disease control, suggesting a favorable therapeutic response and a potential association with overall immune activity.

The coexistence of IgG4-RD and SLE has rarely been reported in previous studies. In 2007, Kobayashi et al. (14) have reported the first case of IgG4-RD overlapping with SLE. Renal involvement of both IgG4 and SLE is particularly uncommon. Zaarour et al. (15) have reported a case of IgG4-related tubulointerstitial nephritis (TIN) with lupus-associated membranous nephritis (MN), which showed a favorable response to high-dose prednisone followed by MMF. Yamamoto et al. (16) have described another case of IgG4-TIN with lupus-induced MN. Although initial treatment with prednisone and MMF achieved clinical improvement, disease control was not sustained following glucocorticoid tapering. Subsequent escalation to belimumab ultimately resulted in sustained disease control.

In this case, we established a diagnosis of IgG4-RD concomitant with SLE and demonstrated tubulointerstitial and glomerular injuries attributed to two distinct disease processes. Fibro-inflammatory tubulointerstitial injury was consistent with IgG4-related TIN based on immunohistochemical findings. However, the mechanism of glomerular nephritis remains to be clarified. Previous studies have reported that IgG4-RD-related glomerular lesions present with or without concomitant TIN, with pathological patterns including MN with or without PLA2R positivity, MsPGN, and endocapillary proliferative glomerulonephritis (17–20).

In this case, MsPGN raised the question of whether it represents a manifestation of IgG4-RKD. Notably, immunofluorescence microscopy revealed a full-house pattern in the mesangial area, suggesting marked complement system activation and differing from previously reported cases of IgG4-RKD. Moreover, persistent heavy proteinuria and hematuria indicated a proliferative lesion that could not be fully explained by IgG4-RKD, as concurrent proteinuria and hematuria have been reported in only 20% of IgG4-RD cases (17). We therefore speculated that the glomerular involvement resulted from extensive immune complex depositions and complement activation.

Considering the presence of positive ANA and DAT, hypocomplementemia, and characteristic immune complex deposition, the MsPGN was ultimately classified as class II lupus nephritis. To the best of our knowledge, this is the first report describing a new pathological pattern in which lupus-associated MsPGN coexists with IgG4-related TIN, suggesting distinct glomerular and interstitial disease processes and supporting the possibility of independent but concomitant pathogenic mechanisms within the kidney.

These two diseases may share potential common pathogenic mechanisms (3, 21). In IgG4-RD, B-cell expansion and differentiation promote the proliferation of IgG4^+^ plasma cells. In SLE, complex autoimmune environments drive the autoreactive B cells toward germinal-center or extrafollicular fates, resulting in the loss of B-cell tolerance and the rapid production of autoantibodies. In addition, previous studies have suggested shared genetic susceptibility between IgG4-RD and SLE, such as Fc gamma receptor 2b (FCGR2B), human leukocyte antigen (HLA)-DRB1, and C4 copy number variation (22–25). Unfortunately, genetic analysis was not performed in this case. In a cohort of 77 patients with IgG4-RKD (17), approximately 30% were ANA positive, whereas only 2% were anti-dsDNA positive. Nevertheless, limited data are available regarding IgG4-RD coexisting with SLE or lupus-like syndromes. In this study, we highlight an overlap syndrome between IgG4-TIN and class II lupus nephritis, emphasizing the need for attention to both clinical and pathological features, as well as potential shared pathogenesis.

From a therapeutic perspective, prednisone combined with HCQ and MMF effectively controlled systemic and cutaneous manifestations during the 8-month follow-up period in this case. We acknowledge that an 8-month follow-up is relatively short for a chronic and potentially relapsing disease. Therefore, long-term close monitoring of the patient’s disease activity and treatment-related adverse effects during follow-up remains essential. In addition to conventional therapies, B-cell-targeted therapies represent a promising therapeutic strategy for both IgG4 and SLE. Agents targeting B-cell activating factor of the tumor necrosis factor (TNF) family (BAFF/BLyS) and a proliferation-inducing ligand (APRIL) have demonstrated efficacy in both diseases, including IgG4-RD coexisting with SLE (16, 26). Furthermore, B-cell depletion represents another potential therapeutic method in disease management. The anti-cluster of differentiation (CD) 20 antibody rituximab has shown efficacy in the treatment of SLE and IgG4-RD (27). CD19-targeted approaches, including CAR T-cell therapy for refractory SLE (5) and inebilizumab for IgG4-RD (28), highlight the vital role of more profound B-cell depletion in disease management.

## Conclusion

This case reports purpura as the initial manifestation of coexisting IgG4-RD and SLE. Renal biopsy revealed concurrent lupus-associated MsPGN and IgG4-related TIN, indicating a novel pathological pattern and emphasizing the importance of renal biopsy in diagnosis. Treatment with prednisone, HCQ, and MMF achieved sustained clinical improvement over an 8-month follow-up period. A review of previous studies has suggested potential shared genetic and immunological mechanisms between IgG4-RD and SLE. In addition to conventional therapeutic methods, B-cell-targeted therapies may offer promising treatment options for such concomitant autoimmune-mediated diseases.

## Data Availability

The datasets presented in this article are not readily available because of ethical and privacy restrictions. Requests to access the datasets should be directed to the corresponding author.
