# Supplementary figures and images for: Purpura as the initial manifestation of IgG4-related disease with concomitant systemic lupus erythematosus: a case report
Source: Front Med (Lausanne). 2026 Jun 22;13:1872392. doi: 10.3389/fmed.2026.1872392 (PMC13333454; doi:10.3389/fmed.2026.1872392)

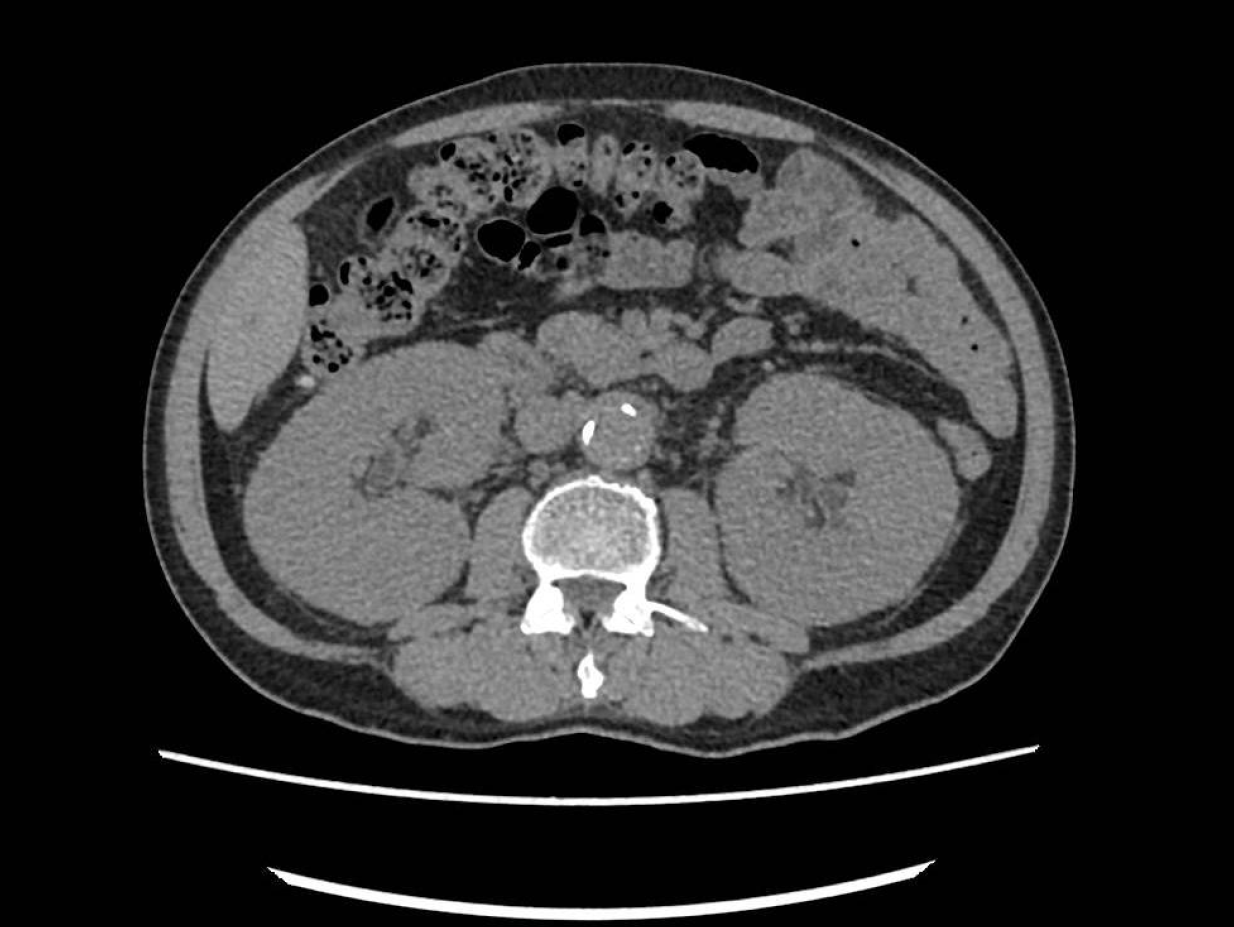

Supplement: Supplementary file 1 [file Image_1.tif]

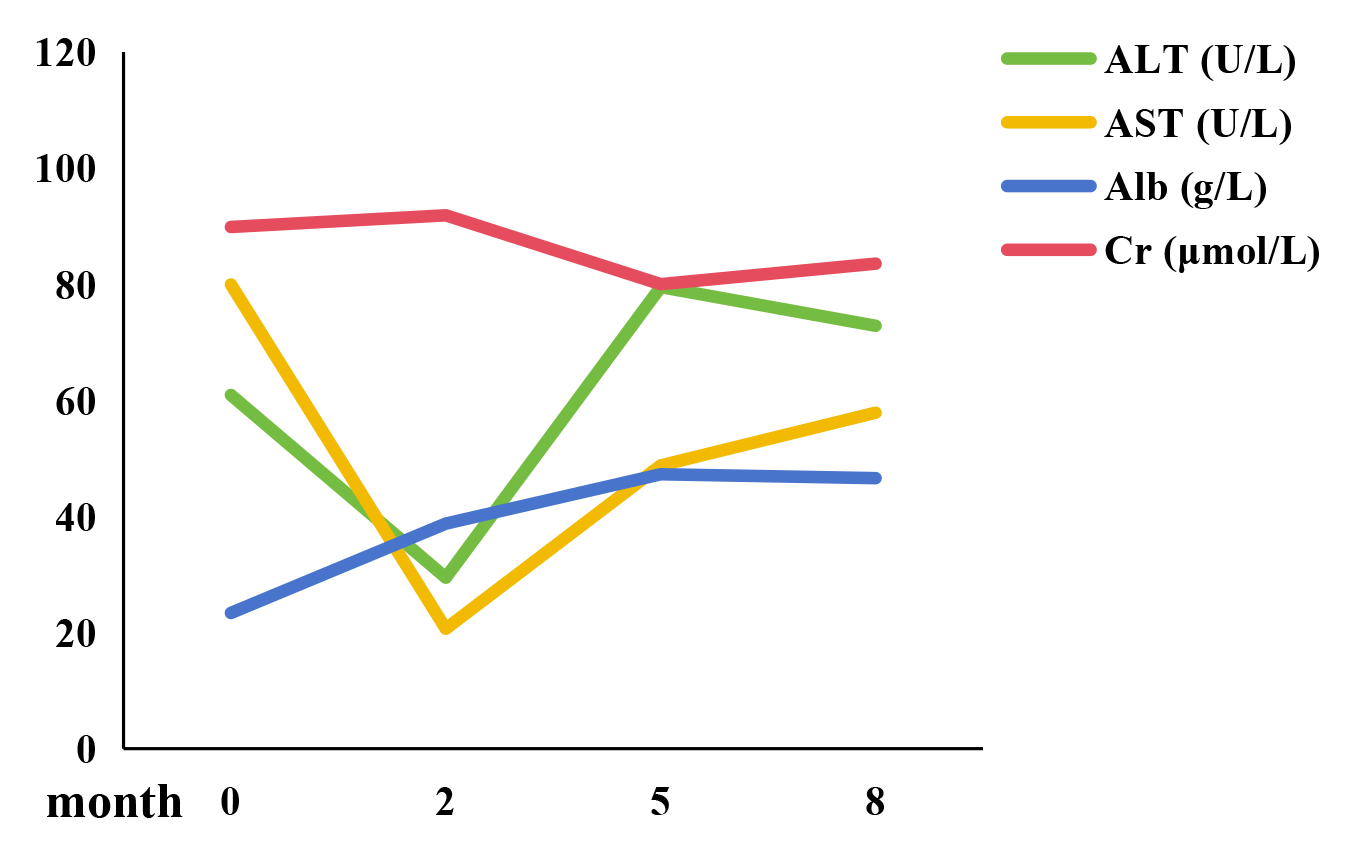

Supplement: Supplementary file 2 [file Image_2.tif]
