# Supplementary material for: Purpura as the initial manifestation of IgG4-related disease with concomitant systemic lupus erythematosus: a case report
Source: Front Med (Lausanne). 2026 Jun 22;13:1872392. doi: 10.3389/fmed.2026.1872392 (PMC13333454; doi:10.3389/fmed.2026.1872392)
Supplement: Supplementary file 3 [file Table_1.docx]

**Table S1. Laboratory data.**

| **Parameters** | **Value** | **Reference range** |
| --- | --- | --- |
| **Urinalysis** |  |  |
| White blood cells (/μL) | 1.98 | 0-10 |
| Red blood cells (/μL) | 25.52 | 0-12 |
| Specific gravity | 1.014 | 1.005-1.03 |
| Protein (g/24h) | 0.83 | 0-0.12 |
| hemoglobin | 1+ | Negative |
| Glucose | Negative | Negative |
| **Complete blood count** |  |  |
| White blood cells (10^9/L) | 6.3 | 4-5.5 |
| Red blood cells (10^12/L) | 4.07 | 4-10 |
| Hemoglobin (g/L) | 111 | 120-160 |
| Eosinophil | 0.09 | 0-0.7 |
| Platelet (10^9/L) | 287 | 100-300 |
| **Coagulation** |  |  |
| Prothrombin time (s) | 13.07 | 9.2-13.9 |
| Activated partial thromboplastin time (s) | 29.96 | 21.2-34.8 |
| Fibrinogen (g/L) | 2.86 | 2-4 |
| **Biochemistry** |  |  |
| Alanine aminotransferase (ALT, U/L) | 60.92 | 0-40 |
| Aspartate aminotransferase (AST, U/L) | 79.96 | 0-40 |
| Albumin (Alb, g/L) | 23.38 | 40-55 |
| Total bilirubin (μmol/L) | 13.64 | 5-21 |
| Indirect bilirubin (μmol/L) | 4.64 | 5-17.6 |
| Direct bilirubin (μmol/L) | 9 | 0-3.4 |
| Blood urea nitrogen (μmol/L) | 6.33 | 2.8-7.1 |
| Creatinine (Cr, μmol/L) | 89.87 | 44-133 |
| Uric acid (mmol/L) | 461.4 | 208-428.4 |

**Table S1. Continued.**

| **Parameters** | **Value** | **Reference range** |
| --- | --- | --- |
| **Infection** |  |  |
| Erythrocyte sedimentation rate (mm/h) | 127.51 | 0-15 |
| Anti-streptolysin O (IU/mL) | 20.66 | 0-250 |
| Hepatitis B surface antigen (IU/mL) | 0 | 0-0.05 |
| Hepatitis B surface antibody (mIU/mL) | 0 | 0-10 |
| Hepatitis B core antibody (S/CO) | 0.33 | 0-1 |
| Hepatitis B e antigen (S/CO) | 1.75 | Not less than 1 |
| Hepatitis B e antibody (S/CO) | 0.16 | 0-1 |
| Hepatitis C virus antibody (S/CO) | 1.65 | 0-1 |
| Hepatitis C virus RNA (IU/mL) | Less than 5×10^2 | Less than 5×10^2 |
| TP-antibody (S/CO) | 0.17 | 0-1 |
| HIV-antibody test (S/CO) | 0.08 | 0-1 |
| T-SPOT | Negative |  |
| **Malignant disease** |  |  |
| Carcinoembryonic antigen (ng/mL) | Less than 1.73 | 0-5 |
| Alpha-fetoprotein (ng/mL) | Less than 2 | 0-8.78 |
| Total prostate-specific antigen (ng/mL) | 0.105 | 0-4 |
| Free prostate-specific antigen (ng/mL) | 0.06 | 0-2.5 |
| Serum immunofixation electrophoresis | Negative |  |
| Serum protein electrophoresis | Negative |  |
| Urine protein electrophoresis | Negative |  |
| κ light chain (g/L) | 24.07 | 0.83-2.24 |
| λ light chain (g/L) | 10.97 | 1.55-4.08 |
| **Endocrine** |  |  |
| Thyroid-stimulating hormone (μIU/mL) | 3.55 | 2.43-6.01 |
| Free triiodothyronine (pmol/L) | 10.98 | 9.01-19.05 |
| Free thyroxine (pmol/L) | 2.4645 | 0.35-4.94 |
