# Supplementary material for: Purpura as the initial manifestation of IgG4-related disease with concomitant systemic lupus erythematosus: a case report
Source: Front Med (Lausanne). 2026 Jun 22;13:1872392. doi: 10.3389/fmed.2026.1872392 (PMC13333454; doi:10.3389/fmed.2026.1872392)
Supplement: Supplementary file 4 [file Table_2.docx]

**Table S2. Diagnostic approaches in the present case.**

| **Criteria** | **2019 EULAR/ACR IgG4-RD classification criteria** | **2020 IgG4-RKD diagnostic criteria** | **2019 EULAR/ACR SLE classification criteria** |
| --- | --- | --- | --- |
| Entry criteria | Diffuse renal enlargement.  Renal biopsy evidence. | Proteinuria and hematuria.  Diffuse renal enlargement. | ANA 1:1000. |
| Serological criteria | IgG4 60.24 g/L.  (11 points) | IgG4 60.24 g/L. | C3 0.47 g/L and C4 0.03 g/L.  (4 points) |
| Pathological criteria | IgG4^+^/IgG^+^ cell ratio > 40% and IgG4^+^ cells > 30/HPF.  (14 points) | IgG4^+^/IgG^+^ cell ratio > 40% and IgG4^+^ cells > 30/HPF.  Storiform fibrosis. | Class II lupus nephritis.  (8 points) |
